# The Knowledge of the Physician

**Published:** 1884-10

**Authors:** 


### BOOK NOTICES.

THE KNOWLEDGE OF THE PHYSICIAN. By Richard Hughes, M. D. Boston: Otis Clapp & Son. 1884.

During his recent visit to America Dr. Hughes delivered a course of lectures at the Boston University School of Medicine. These are now given to the public in book form by Messrs. Clapp & Son.

All of our readers who are acquainted with the writings of Dr. Hughes remember most agreeably his charming style, and perhaps equally forcibly regret that he teaches such poor Homœopathy.

The following topics are treated of: The Knowledge of Life; The Knowledge of Health; The Knowledge of Disease; The Knowledge of Medicines; Pyrexia and Anti-Pyretics; Rheumatism and the Anti-Rheumatics; Cerebral Localization and Drug Action; The Future of Pharmaco-Dynamics. These headings show the nature and extent of the lectures. We need only add that the subjects are considered after the well-known peculiar ideas of the lecturer. About the only text-book of Therapeutics the lecturer appears to admire is one on *Pharmaco-Dynamics*, by a certain Richard Hughes, M. D.; all else is chaff!!

